# The Effect of Background Music on Inhibitory Functions: An ERP Study

**DOI:** 10.3389/fnhum.2018.00293

**Published:** 2018-07-23

**Authors:** Anja Burkhard, Stefan Elmer, Denis Kara, Christian Brauchli, Lutz Jäncke

**Affiliations:** ^1^Department of Neuropsychology, Psychological Institute, University of Zurich, Zurich, Switzerland; ^2^Dynamics of Healthy Aging, University Research Priority Program (URPP), University of Zurich, Zurich, Switzerland

**Keywords:** EEG, Go/NoGo, event-related potentials, background music, arousal, inhibition

## Abstract

The influence of background music on cognitive functions is still a matter of debate. In this study, we investigated the influence of background music on executive functions (particularly on inhibitory functions). Participants completed a standardized cued Go/NoGo task during three different conditions while an EEG was recorded (1: with no background music, 2: with relaxing, or 3: with exciting background music). In addition, we collected reaction times, omissions, and commissions in response to the Go and NoGo stimuli. From the EEG data, event-related potentials (ERPs) were calculated for the Go and NoGo trials. From these ERPs, the N2 and P3 components were specifically analyzed since previous studies have shown that these components (and particularly the Go-NoGo difference waves) are strongly associated with inhibitory functions. The N2 and P3 components of the difference waves (N2d and P3d) were used for statistical analyses. The statistical analyses revealed no differences between the three conditions in terms of amplitudes and latencies of the N2d and P3d components. In addition, reaction times, omissions, and commissions were comparable across all conditions. Our results suggest that in the context of this paradigm, music as background acoustic stimulation has no detrimental effects on the performance of a Go/NoGo task and neural underpinnings.

## Introduction

Today, music is easily available and frequently consumed. In addition, the scientific interest in studying the influence of music listening on different psychological functions has substantially increased. Based on surveys conducted by North et al. (2004) and Krause et al. (2015), respondents reported that music is important to them and that they listened to musical pieces at least 1 h per day. One possible reason for such a high prevalence of music listening in everyday life might be related to the fact that music has previously been shown to be used to regulate emotions (Thoma et al., 2006). Furthermore, recent advances in technology have put music at the forefront of people’s common practices, with instant access to countless musical libraries (North et al., 2004).

The influence of background music on several activities and on cognition in general is still a matter of debate. Most of the work published so far has focused on exposure to music in a work-related setting or while engaging in routine activities such as driving a car (Jäncke et al., 1994; Furnham and Bradley, 1997). The influence of background music on the performance of school-related skills and academic tasks has also been investigated (Fogelson, 1973; Etaugh and Ptasnik, 1982; Crawford and Strapp, 1994; Hallam et al., 2002; Doyle and Furnham, 2012). Although the results are mixed, most studies revealed a detrimental influence of background music on task performance. Furthermore, other cognitive functions such as verbal learning have not been found to be influenced by background music (Jäncke and Sandmann, 2010). Other studies have revealed better performance in paper folding and cutting tasks during acoustic background stimulation as opposed to silence (Nantais and Schellenberg, 1999; Thompson et al., 2001). A positive influence of background music was also found in the popular study by Rauscher et al. (1993) where performance of an abstract/spatial reasoning task was temporally enhanced after listening to music. It is also known that music positively influences memory performance (Peynircioğlu et al., 1998; Platel, 2005; Eschrich et al., 2008). This is because of its ability to evoke strong emotions which in turn increase memory performance (Jäncke, 2008). A positive influence of background music has also been found for emotional reactions and sports achievements (Kämpfe et al., 2011). Conversely, some studies have provided evidence that performance is better without than with background music (Furnham and Bradley, 1997; Sousou, 1997). Furthermore, some studies have concluded that music has detrimental effects on completing memory tests, reading comprehension (Furnham and Bradley, 1997), or driving a car in a computer-simulated setting (Jäncke et al., 1994). A meta-analysis by Kämpfe et al. (2011) also revealed that background music has a negative influence on reading comprehension and on a variety of memory tasks. In summary, the findings of studies investigating the influence of background music on several tasks are mixed, but most of them reported that background music exerts a detrimental influence on cognitive functions.

Along with the discussion on how background music influences the completion of a given task, there are several studies focusing on additional phenomena. Sousou (1997) showed that it is possible to induce moods with music. She also suggested that music may interfere with the learning process if the learning material does not correspond to the kind of music played in the background, for example, listening to happy music while trying to memorize sad lyrics. Furthermore, Thompson et al. (2001) revealed that the performance of certain tests of spatial abilities improves if a music piece is applied, which leads to increased arousal and positive affect. A study by Jäncke and Sandmann (2010) reported an increase of cortical activation during a verbal learning task while background music was played in contrast to a noise condition. However, this effect does not seem to be an effect of arousal because no significant differences in arousal were found between the music and noise conditions. Therefore, these results might indicate that increased effort is needed to suppress background music while executing such a task (Jäncke et al., 2014), which in turn might be reflected in neurophysiological measurements.

Little is known about the influence of background music on executive functions. A study of Zuk et al. (2014) reveals that certain executive skills might benefit from music training, but concerning non-musicians, less is known about the influence of background music on executive functions such as inhibition, conflict monitoring, and cognitive control. Almost all daily activities, such as shopping and working, depend on executive functions which are needed for administrating cognitive control. According to Squire et al. (2012), the term “cognitive control” describes the ability to anticipate possible outcomes and initiate appropriate actions to reach a given goal. Cognitive control includes a whole subsystem of processes, such as initiating, inhibiting, shifting, monitoring, guiding, planning, and simulating possible outcomes (Gazzaniga and Mangun, 2014). Inhibitory mechanisms are typically examined by using a Go/NoGo task (Falkenstein et al., 1999). In such a task, participants are instructed to execute a response to specific stimuli (Go) and to withhold the response (NoGo) if other stimuli are presented. A variant of that task is the so-called and frequently used visual continuous performance task (VCPT) (Kropotov et al., 2011, 2016, 2017; Meier et al., 2012; Kropotov and Ponomarev, 2015).

Two event-related potential (ERP) components which are associated with response inhibition are elicited during Go/NoGo tasks: a negative shift between 200 and 350 ms (NoGo-N2) (Folstein and Van Petten, 2008) and a positive shift between 300 and 500 ms (NoGo-P3) (Falkenstein et al., 1999). These components are obtained by computing difference waves of the ERPs by subtracting the Go from the NoGo trials (revealing the difference components for N2 and P3: N2d and P3d) (Falkenstein et al., 1999). Results from ERP studies using sequential matching tasks suggest that the frontal N2 component can be attributed to mismatch detection (Suwazono et al., 2000; Wang et al., 2003). In addition, Enriquez-Geppert et al. (2010) reported that the N2 elicited by a combined Go/NoGo and stop-signal task reflects conflict-related processes. Increased NoGo-N2 amplitudes have also been found if the participants were forced to react quickly (Jodo and Kayama, 1992). Furthermore, Azizian et al. (2006) provided evidence that NoGo stimuli which are similar to Go stimuli elicit larger N2 amplitudes because they trigger a preparation for a false response that has to be suppressed. Otherwise, the P3 component includes two subcomponents that show different topographies: the P3a has a frontally distributed maximum on the scalp, whereas the maximum of P3b lies over parietal scalp sites (Folstein and Van Petten, 2008). The earlier subcomponent is usually associated with turning one’s attention to significant or non-expected events, whereas the latter seems to reflect working memory processes (Folstein and Van Petten, 2008). In a more general view, the P3 represents motor and/or action inhibition (Enriquez-Geppert et al., 2010; Kropotov et al., 2016, 2017).

Based on the fact that cognitive control processes are omnipresent in most of our daily activities and by taking into account the essential role of music in our culture, the effects of background music on cognitive performance deserve greater attention. Therefore, in the present study, we collected behavioral and electroencephalographic (EEG) data while participants performed the VCPT three times in randomized order. The task was performed with no music (NM) in the background, while listening to a relaxing song (RLX), and while being exposed to an exciting one (EXC). The musical pieces were selected to test whether different degrees of arousal differentially affect inhibitory functions.

So far, most of the published studies have used a between-subject design. Here, we chose a within-subject design to compare the effect of the experimental manipulations on the same participants. Furthermore, we are not aware of studies that have examined the influence of background music on inhibitory mechanisms in a controlled setting.

Based on Thompson et al. (2001), we assumed that task performance would improve with increased arousal. Therefore, we expected that the RLX condition would lead to worse performance than the EXC condition. Furthermore, we hypothesized that the task performance and the neurophysiological measurements of the two musical conditions would differ from our NM condition in an unspecific direction since there are mixed results regarding the influence of background music on task performance (Furnham and Bradley, 1997; Sousou, 1997; Nantais and Schellenberg, 1999; Thompson et al., 2001). We also investigated whether an increase in cortical activation, as was found by Jäncke and Sandmann (2010), would take place during a cognitive control task and if it would be reflected by ERP components. The increased cortical activation should be reflected by increased NoGo-N2 and NoGo-P3 amplitudes during the two musical conditions (RLX and EXC). Furthermore, better performance is expected to be reflected by shorter reaction times and fewer errors in task completion.

## Materials and Methods

### Participants

In the present study, we examined 25 (17 female) volunteers. Participants were screened for mental disorders, medication, drug, and alcohol abuse. Five participants reported present or past neurological, psychiatric, or physiological disorders and were therefore excluded from further analysis. One participant was excluded due to extensive EEG artifacts. The participants were between 20 and 30 years old (average 23 years, SD = ± 2.87). All participants were right-handed (Annett, 1970; Bryden, 1977) and had at least a certificate from grammar school or a bachelor’s degree (years of education: 12.9 ± 1.4). All participants had a normal or corrected visual acuity and reported no hearing impairments. The mother tongue of all participants was German. All participants denied having received musical education for the last 5 years. The reason why we focused on non-musicians was to avoid having musical experts in the sample who could be more affected by the music. The participants provided informed consent and were paid for their attendance in the study. The study was approved by the Ethics Committee of the University of Zurich in accordance with the Declaration of Helsinki.

### Experimental Design

**FIGURE 1:**
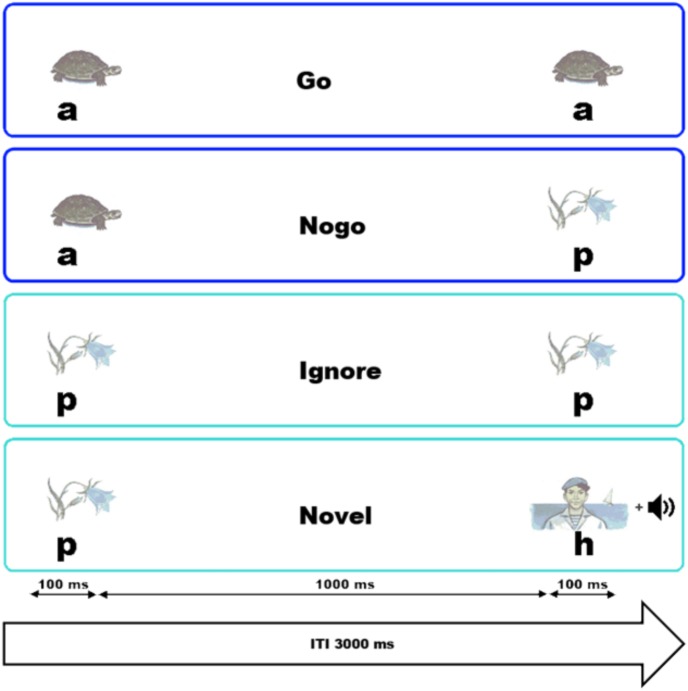
Graphical representation of the tasks and their time dynamics. The instruction in the Go trials was to press a button with the right index finger if two animal (a) pictures were presented in succession. The participant was asked to withhold the reaction in a NoGo trial, when an animal and a plant (p) picture were shown sequentially. For the Ignore trials, two plant pictures were presented in succession and no action was required. In the Novel trials (i.e., no action was required from the participant), a plant followed by a picture of a human (h) and a novel sound were presented.

To examine the influence of background music on inhibitory functions, the VCPT was used combined with background music. The entire procedure lasted for approximately 75 min. In each trial of the VCPT, two pictures were presented in succession. The stimulus material comprised a total number of 60 pictures which had similar luminosity and size and were divided into three different stimulus categories, namely 20 unique pictures of animals (a), 20 of plants (p), and 20 of humans (h). Their actual sequential order gave the participants the cue to react as fast and as precisely as possible or to suppress the motor reaction. The stimuli pairs were a–a, a–p, p–p, or p–h, as illustrated in **Figure 1**.

For the Go trials, pairs of physically identical animal pictures (**Figure 1**, a–a) were presented, and the participant had to press a button with the right index finger. An animal picture followed by a plant picture represented the NoGo (**Figure 1**, a–p) trial. In NoGo trials, the participants had to withhold button pressing. In the Ignore trials, a picture of a plant was followed by a second physically identical plant picture (**Figure 1**, p–p); no response was required. Likewise, in Novel trials no response was required. The Novel trials consisted of a plant picture followed by a human picture (**Figure 1**, p–h). In summary, whenever an animal appeared as the first picture, a response had to be prepared in case a second animal would appear. As soon as a plant was presented as the first stimulus, no motor response was required. To guarantee a certain level of alertness, the pictures of humans were presented together with a novel acoustic stimulus. Those acoustic stimuli comprised 20 randomly presented tone fragments at different frequencies with a length of 100 ms. The trials were presented in a randomized order with an inter-stimulus interval of 1000 ms and an inter-trial interval of 3000 ms. The pictures were presented for a duration of 100 ms. A block consisted of 100 trials, and for each block a unique set consisting of 5 animal, 5 plant, and 5 human pictures was selected. The probability for each trial category to occur was equal. Four blocks were presented for each run of the VCPT. This resulted in a total of 400 visual trials and a duration of approximately 20 min per run. Before the start of the experiment, the task was practiced by the participants. The VCPT was presented using the software PsyTask (Kropotov et al., 2016, 2017).

### Experimental Conditions

The VCPT was performed three times under different conditions. In one condition, the participants had to complete the task without background music (NM). In the other two conditions, the execution of the task was accompanied by instrumental background music. The music pieces were chosen based on intensity ratings taken from the publication by Jäncke et al. (2014) and were thought to elicit states of excitation and relaxation, respectively. John Williams and William Ross’ “Reunion of Friends” served as a soothing piece of music due to its low subjective intensity and was used for the relaxing condition (RLX). The song had a duration of 5 min and 9 s. A stimulating piece and second condition (EXC) was the song “The Planets – Jupiter, the Bringer of Jollity” from Gustav Holst due to its high intensity rating. The duration of the song was 7 min and 36 s. Details of the intensity ratings for both musical pieces can be found in Jäncke et al. (2014). The conditions were presented in randomized order. The musical pieces were looped until a run of the VCPT was accomplished. The audio editor and recorder Audacity (Version 1.3 Beta, The Audacity Team, United States) was used to align the songs for volume adjustment to provide the same amplification across both compositions. Acoustic irradiation was undertaken at a moderate hearing volume of 70 dB. The light was kept on during the entire experiment.

### Behavioral Measurements

A short arousal and valence in-house questionnaire was filled out by participants after each condition. In particular, the subjective arousal level was rated on a 7-point Likert’s scale ranging from 1 (not aroused at all) to 7 (highly aroused). The same procedure was adopted for the mood evaluation ranging from 1 (very sad) to 7 (very happy). Also, the valence was rated for the two songs, namely how much the participants liked them. This scale ranged from 1 (no liking at all) to 7 (liked it very much).

### Performance Measurements

Reaction times were calculated as the time elapsed between the onset of the second picture and the participant’s button press. Trials with a reaction time within 200–1000 ms after onset of the second stimulus were used for the averages (Kropotov et al., 2011). Also omission errors, namely, the failure to respond in Go trials, and false alarm rates (i.e., participant failed to suppress a response to NoGo trials) were collected. Mean reaction times, omission errors, and false alarm rates were evaluated separately for every participant and for the experimental conditions NM, RLX, and EXC.

### EEG Recording

The EEG measurement was carried out by using Comby EEG Caps with 19 AgCl electrodes, with a Neuroamp^®^x23 amplifier system, both manufactured by BEE Medic GmbH (BEE Medic GmbH, Germany). EEGs were recorded using the ERPrec recording software (Version 2.0.x, BEE Systems, Germany). The electrodes Fp1, Fp2, F7, F3, Fz, F4, F8, T3, C3, Cz, C4, T4, T5, P3, Pz, P4, T6, O1, and O2 were placed according to the international 10–20 system. The signal was digitized with a sampling rate of 250 Hz and an online high-pass filter of 0.16 Hz was applied. Impedances were kept below 20 kΩ using conductive gel and the online reference was Cz. The musical pieces were presented binaurally via Bose Companion 2 Series III external multimedia computer speakers. Participants were asked to blink before each measurement, to bite their teeth, and to produce saccades to demonstrate to them the effects of such movements during the experiment. Thereupon, the participants were asked to sit as relaxed as possible during the entire procedure and to omit the aforementioned actions as well as frowning.

### EEG Pre-processing

The pre-processing of the data was done in WinEEG (Version 2.84.44, Mitsar, Russia), MatLab (Version R2015b, MathWorks, United States), and Brain Vision Analyzer (Version 2.1, Brain Products, Germany). The initial stages of the pre-processing were done in the WinEEG software. The data were offline re-referenced to the so-called “average montage.” This montage includes a bandpass filter with a low-cut of 0.53 Hz and a high-cut at 50 Hz. It also applies a notch filter at 45–55 Hz. Eye movement artifacts were corrected by using individual independent component analysis by removing the corresponding independent components based on the individual activation curves (Vigário, 1997; Jung et al., 2000; Li et al., 2006). In a further step, segments which contained excessive amplitudes or frequencies were marked and rejected. For exclusion the following thresholds were applied: 100 μV for non-filtered EEG, 50 μV for 0–1 Hz filtered (slow waves), and 35 μV for 20–35 Hz filtered (fast waves) EEG. A total of 200 ms before and after each event were excluded. Additionally, artifacts were excluded after manual inspection of the entire EEG data.

**FIGURE 2:**
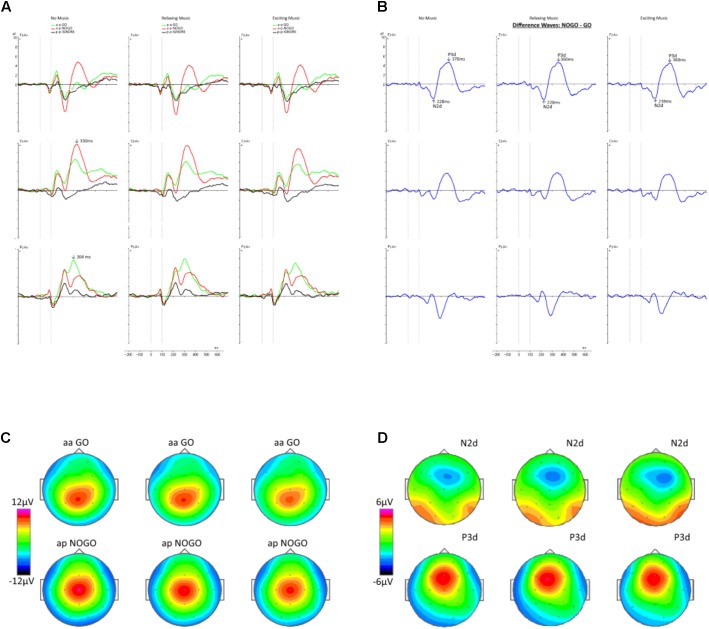
**(A)** The grand average ERP waveforms in the three conditions are shown for the midline electrodes Fz, Cz, and Pz. The onset of the second stimulus is at time point zero, whereas the second dashed line represents its offset. The green waveforms show the average ERPs for the Go trials, the red ones represent the NoGo trials, and the black curves indicate the Ignore trials. **(B)** The difference waves corresponding to **(A)** are depicted for the different conditions. Maximal amplitudes of the N2d and P3d components are indicated by arrows. **(C)** Topographies in the top row were computed for Go trials and the bottom row represents the NoGo trials. **(D)** Topographical maps of the N2d (top row) and P3d (bottom row) components.

Afterward, averages for Go-ERPs and NoGo-ERPs for each condition and subject were computed, starting from the presentation of the second stimulus. A baseline correction from -200 ms to the onset of the second stimulus was applied to the ERPs. Furthermore, difference waves were computed by subtracting the average Go-ERP from the average NoGo-ERP for midline electrodes (Fz and Cz) for each condition. The values of the difference waves at electrode Fz and Cz were exported, converted with MatLab, and further processed via Brain Vision Analyzer software. We focused on these two electrodes because previous studies have shown that the components of interest, namely, N2 and P3, are most pronounced at Fz and Cz (Kropotov et al., 2011, 2016; Alahmadi, 2017). Especially, the N2 and P3 components measured at frontal electrodes reflect neurophysiological responses associated with cognitive control like conflict monitoring and action inhibition (Kropotov et al., 2016, 2017). We conducted semi-automatic peak detection for the N2 and P3 components of the difference waves for every subject and condition. From now on, whenever referring to the N2 and P3 components, we will use the terms N2d and P3d, respectively, to emphasize that they are obtained from difference waves (Falkenstein et al., 2002). Based on the grand average waveforms (**Figure 2A**), global maxima were detected in the range of 200–350 ms for the N2d waveform (Folstein and Van Petten, 2008), and from 300 to 500 ms for the P3d waveform (Falkenstein et al., 1999). The values for the N2d and P3d components of the average difference wave ERPs were compared for amplitude and latency among the conditions. The amplitudes were evaluated by selecting the peak amplitude and computing the mean amplitude over a time window of 50 ms. The latency onsets were measured with fractional peak latency. This method relies on the identification of the time point where the amplitude of the waveform reaches a given percentage of the peak amplitude (Luck, 2014). Here, we used the so-called “50% peak latency” because in most cases it has the highest reliability (Kiesel et al., 2008).

### Statistical Analysis

All statistical analyses were conducted by using the software package SPSS (Version 22, IBM, United States). For the subjective arousal level, three Wilcoxon signed-ranks tests were calculated. In particular, we compared both conditions with background music to our NM baseline condition as well as the EXC and RLX conditions. The same was done for the mood ratings of the participants. A further Wilcoxon signed-ranks test was calculated for the valence ratings of the two different songs. The *p*-values were Bonferroni–Holm-corrected (Holm, 1979). Omission errors, false alarm rates, and reaction times were compared between the different conditions by means of univariate repeated measures analyses of variance (ANOVA). We also conducted four two-way repeated measures ANOVAs for the ERP components of interest. The first within-subject factor comprised three condition levels (i.e., NM, RLX, and EXC). The second within-subject factor represented the electrode sites and had two levels (Fz and Cz). Before computation of the ANOVAs, we checked whether the data fulfilled all requirements for conducting these analyses. In case of heteroscedasticities, Greenhouse-Geisser-corrections were applied for the ANOVA results. We computed two ANOVAs for mean amplitude values (N2d and P3d) and two for peak latencies. A *p*-value of 0.0125 (in the context of two-tailed testing) was considered significant according to Bonferroni–Holm correction as a consequence of multiple comparisons. Beside *p*-values, we also report effect size measures. For ANOVAs, we report the partial eta-squared (η^2^), and effect sizes of Wilcoxon signed-ranks tests are given in *r*. Both effect sizes are interpreted as suggested by Cohen (1988, 1992).

## Results

### Behavioral Data

Three Wilcoxon signed-ranks tests were computed for evaluating the arousal data. Both arousal ratings with background music differed significantly from the condition without background music. The arousal rating in the RLX condition was significantly higher (median = 4) compared to the NM (median = 2) condition (*z* = -2.45, *p* = 0.013, *r* = 0.56). In addition, the arousal ratings for EXC were higher compared to the NM condition (*z* = -2.39, *p* = 0.014, *r* = 0.55). A further Wilcoxon signed-ranks test yielded no significant differences among the experimental conditions, namely the difference between RLX and EXC (*z* = -1.23, *p* = 0.305, *r* = 0.28). The three Wilcoxon signed-ranks tests computed for the mood ratings revealed no differences between the conditions, and no differences were found between the NM (median = 5) and the RLX (median = 5) conditions (*z* = -1.67, *p* = 0.188, *r* = 0.38). We did not reveal differences between the NM and the EXC (median = 5) conditions (*z* = -1.41, *p* = 0.312, *r* = 0.32), and also the RLX and the EXC conditions were comparable (*z* = -0.38, *p* = 1.0, *r* = 0.09). The Wilcoxon signed-ranks test computed for the valence ratings of the relaxing (median = 5) and the exciting (median = 5.0) songs did not reveal significant differences (*z* = -0.79, *p* = 0.479, *r* = 0.18).

### Performance Data

**Table 1** shows that the individual computations for omission rates, false alarms, and reaction times did not reach a level of significance, irrespective of the musical condition that the participants were exposed to. A univariate repeated measure ANOVA revealed that participants’ reactions to the stimuli across the various conditions did not differ in terms of reaction times [*F*(2,36) = 0.05, *p* = 0.947, partial η^2^ = 0.003], omission errors [*F*(1.46,26.32) = 0.78, *p* = 0.432, partial η^2^ = 0.041], or false alarm rates [*F*(2,36) = 1.44, *p* = 0.250, partial η^2^ = 0.074].

**Table 1:** Performance data of the three conditions.

Condition	False alarms in NOGO trials	Omissions in GO trials	Reaction time (ms) in GO trials

	(Mean frequency ± SE)	(Mean frequency ± SE)	(Mean duration) ± SE)
**NM**			
No Music	0.68 ± 0.20	3.00 ± 0.65	313.68 ± 13.78
**RLX**			
Relaxing Music	0.74 ± 0.21	3.79 ± 0.84	315.53 ± 15.62
**EXC**			
Exciting Music	0.42 ± 0.14	3.53 ± 0.75	314.05 ± 13.79


*The means and standard errors are shown for false alarms, omission errors, and reaction times. For the false alarms and omissions, the frequencies of occurrence are shown. The NM condition is represented in the first row, followed by the RLX condition in the middle row, and the EXC condition in the bottom row.*

### EEG Data

In **Figure 2A**, the grand averages of the Go, NoGo, and Ignore trials are displayed for the different conditions. A maximum deflection at the electrode Pz with a latency of 304 ms is shown for the Go trials. The topographic maps (**Figure 2C**) reveal a parietal-central distribution. For the NoGo trials, a maximum was identified at electrode Cz with a latency of 330 ms and a central scalp distribution. The corresponding difference waves are depicted in **Figure 2B**. In accordance with the previous studies (Kropotov et al., 2011, 2016; Alahmadi, 2017), the minimum and maximum were located around the Fz and Cz electrode (**Figure 2D**). The minimum voltage was found around 230 ms with a fronto-central distribution. Otherwise, the maximum was found at about 360 ms and reflected by a fronto-central topography.

**FIGURE 3:**
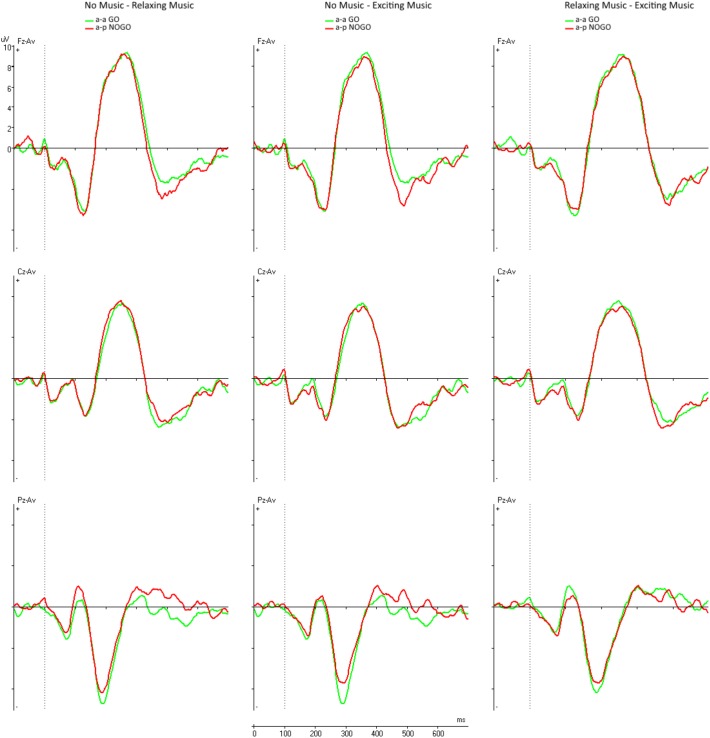
Difference waves at midline electrodes Fz, Cz, and Pz across the conditions. Left column = NM and RLX, middle column = NM and EXC, right column = RLX and EXC.

**Figure 3** shows the paired comparisons of the difference waves across the different conditions. The left overlay displays the comparison between the NM and RLX conditions. The middle column shows the NM compared to the EXC condition. The right column shows the comparison between RLX and EXC.

Two-way repeated measure (3 × 2) ANOVAs with the within-subject factors Music (NM, RLX, EXC) and Electrode (Fz, Cz) for the amplitudes and latencies of N2d and P3d did not reveal any significant main effect for the factor Music [N2d amplitude: *F*(2,36) = 0.11, *p* = 0.892, partial η^2^ = 0.006; N2d latency: *F*(2,36) = 0.02, *p* = 0.984, partial η^2^ = 0.001; P3d amplitude: *F*(2,36) = 0.41, *p* = 0.666, partial η^2^ = 0.022; and P3d latency *F*(1.46,26.31) = 1.99, *p* = 0.165, partial η^2^ = 0.100] and its interaction with the Electrode factor. As **Table 2** shows, there was only a main effect for Electrode with respect to the amplitude of the N2d component [*F*(1,18) = 7.07, *p* = 0.016, partial η^2^ = 0.282], which was qualified by larger amplitudes at Fz compared to Cz (**Table 3**).

**Table 2:** Results of the two-way repeated measures ANOVAs for the N2d and P3d amplitudes and latencies.

		Sum of squares	df	Mean square	*F*	*p*	partial η^2^
**N2d Amplitude**							
	music	0.39	2	0.20	0.11	0.892	0.006
	error(music)	61.78	36	1.72			
	electrodes	65.42	1	65.42	7.07	0.016	0.282
	error(electrodes)	166.68	18	9.26			
	music^∗^electrodes	0.48	2	0.24	0.29	0.751	0.016
	error(music^∗^electrodes)	29.73	36	0.83			
**P3d Amplitude**							
	music	2.88	2	1.44	0.41	0.666	0.022
	error(music)	126.21	36	3.51			
	electrodes	34.66	1	34.66	5.55	0.030	0.236
	error(electrodes)	112.51	18	6.25			
	music^∗^electrodes	0.74	2	0.37	0.42	0.658	0.023
	error(music^∗^electrodes)	31.43	36	0.87			
**N2d Latency**							
	music	22.74	2	11.37	0.02	0.984	0.001
	error(music)	25955.93	36	721.00			
	electrodes	11400.00	1	11400.00	3.74	0.069	0.172
	error(electrodes)	54816.00	18	3045.33			
	music^∗^electrodes	394.95	1.36	290.57	0.59	0.500	0.032
	error(music^∗^electrodes)	12101.05	24.47	494.60			
**P3d Latency**							
	music	3585.33	1.46	2452.90	1.99	0.165	0.100
	error(music)	32373.33	26.31	1230.46			
	electrodes	1417.58	1	1417.58	0.70	0.413	0.038
	error(electrodes)	36291.09	18	2016.17			
	music^∗^electrodes	140.84	2	70.42	0.08	0.919	0.005
	error(music^∗^electrodes)	30084.49	36	835.68			


*Sum of squares, degrees of freedom (df), mean squares, F-values (F), p-values (p), and the partial eta squared (*η^2^*) are listed.*

**Table 3:** N2d and P3d mean amplitudes and 50% peak latency values for the Fz and Cz electrodes.

Fz and Cz electrodes				

Condition	N2d Amplitude (μV)	P3d Amplitude (μV)	N2d Latency (ms)	P3d Latency (ms)
	Mean ± SD	Mean ± SD	Mean ± SD	Mean ± SD
**NM**				
Fz	-3.00 ± 1.66	5.24 ± 3.20	202.21 ± 30.88	350.32 ± 36.21
Cz	-1.59 ± 2.97	4.16 ± 3.76	219.47 ± 57.94	354.42 ± 30.78
**RLX**				
Fz	-3.02 ± 1.67	4.95 ± 2.87	201.16 ± 29.77	355.47 ± 32.42
Cz	-1.32 ± 2.30	4.03 ± 3.64	218.63 ± 39.31	363.05 ± 43.86
**EXC**				
Fz	-3.00 ± 1.65	4.97 ± 2.75	198.21 ± 33.35	361.37 ± 34.65
Cz	-1.57 ± 2.38	3.66 ± 2.94	223.47 ± 53.14	370.84 ± 47.99


## Discussion

### General Discussion

In this work, we examined the influence of different kinds of background acoustic stimulation on the performance (reaction times, omission errors, and false alarm rates) when working on a standard and frequently used Go/NoGo task. In addition, we recorded the associated neurophysiological responses (amplitudes and latencies of the N2d and P3d components) reflecting the neural underpinnings of Go/NoGo performance. With the N2d and P3d components, we are in the position to disentangle at least two different processes underlying executive control. The frontal N2d is associated with conflict monitoring while the frontal P3d is a well-known and stable proxy for neurophysiological processes associated with inhibition of actions and cognitions (Jodo and Kayama, 1992; Falkenstein et al., 1999; Azizian et al., 2006; Folstein and Van Petten, 2008; Kropotov et al., 2011, 2016, 2017). The task was conducted once without music played in the background and twice with background music (i.e., RLX and EXC). To increase the ecological validity, entire musical pieces were played instead of simple tones, noises or fragments of songs. Our first hypothesis was that the performance would improve with exciting music compared to relaxing music. Second, we hypothesized that both musical conditions would differ from the condition without acoustic background stimulation. Third, we expected an increase in cortical activation in the musical conditions, which should be reflected by increased N2d and P3d amplitudes. However, none of these hypotheses could be confirmed. In particular, we did not find differences in performance between the NM, RLX, and EXC conditions, nor differences in ERP modulations.

### Behavioral Results

When people have the possibility to select their own music, they will usually choose the pieces they like most. However, in our study, we did not take into account personal preferences but rather focused on previously tested material which has been shown to induce different degrees of activation. Therefore, it was beyond the focus of this study to evaluate putative relationships between music preference and performance, and we rather examined the influence of background music in general and also the influence of arousal and valence on cognitive performance. This experimental approach is particularly powerful in that it enables to circumvent several confounding factors caused by different musical features (e.g., musical genre, rhythm, beats per minute) instead of experimental manipulation. Otherwise, a disadvantage of this approach is that some ecological validity has to be sacrificed because people usually choose their own soundtrack. Thereby, it is important to mention that to allow participants to take control over the music they are listening to leads to contentment and further motivation to listen (Krause et al., 2015).

The instrumental pieces used in the present study have already been used by Jäncke et al. (2014). According to Jones et al. (2000), it is unlikely that a song with vocals will cause more distraction, because the key factor for distraction is to what extent changes in the auditory stimulus occur. This finding concerns solely a memory task with lists of letters. However, this finding was supported by a study of Jäncke et al. (2014) which did not find an effect on verbal learning concerning music with or without vocals. One condition of that study consisted of learning German words while listening to music with German vocals. The task material of our study comprised only pictures that had no similarity to the applied auditory stimuli. Considering that even similar stimuli cause no distracting effect, we can assume that choosing two vocal pieces instead of instrumental pieces would not affect the degree of distraction they might cause.

In our study, a clear increase in the arousal rating was observed between the no background music condition and the two musical conditions, irrespective of the kind of music that was played. This indicates that music might have a lifting effect on the arousal level in general if it is played in the background. It is also possible, that the selected music was not suitable for inducing a sufficient level of arousal, which in turn might be necessary to influence the performance of task completion in any direction. However, no differences were seen in the arousal ratings between relaxing and exciting background music. One possible way to avoid this problem is to let the participants choose their own music, which is commonly used for daily activities and is rated as relaxing or exciting. The fact that the music pieces were looped until an entire VCPT block was completed should not have influenced the data in some direction. In fact, a previous EEG study (Jäncke et al., 2015) focusing on the electrophysiological effects of music repetition did not reveal EEG changes with the multiple presentation of the same musical piece.

In the present study, we did not give any instructions concerning how the background stimulation had to be treated. This may have resulted in a situation where the participants simply ignored the music. Therefore, only a small amount of cognitive capacity was allocated to it. We decided against giving any instructions because in daily life people also do not follow any instructions about how to deal with the music they are listening to while completing a given task. Our results, as well as those of Jäncke et al. (2014), lead to the assumption that if pre-selected music is used to induce a certain level of arousal in a specific direction, it has to be kept in mind that the manipulation might not show the desired effects.

The performance data (reaction times, omission errors, and false alarm rates) did not differ among the conditions. In general, very few errors (omission and commission) occurred during task completion. Therefore, we can conclude that the participants took the given task seriously and completed it with concentration. This seems to be even more the case because the entire recording took approximately 75 min, and therefore signs of fatigue would be very likely to occur. On the other hand, the low error rates could also indicate that the task was too easy and therefore music did not represent any interference at all. This might be the case because, first, a task with a higher level of difficulty is expected to activate more frontal processes (Stuss and Alexander, 2000). Second, more resources would be allocated to such a task and therefore fewer resources would be available for the suppression of the distracting stimuli. Perhaps during the completion of a more difficult task, as for example, the task shifting Schuch and Koch (2003) used in their experiments, which would require more cognitive resources, our musical pieces would represent a stronger interference. This would be in agreement with the assumption of Furnham and Bradley (1997) that the more complex the task, the more negative the influence of background music is to be expected. Anyhow, the main aim of this study was to use a task that is quite well established in the literature (Kropotov et al., 2011, 2016, 2017; Kropotov and Ponomarev, 2015). Another criterion for choosing this task was to keep the task complexity as close as possible to the tasks experienced in daily life. For example, in general, we are able to drive a car properly without violating traffic regulations. Also, the complexity of the background music was not considered in our study. According to Furnham and Allass (1999), one can assume that more complex background music could have a stronger influence on the performance of the main task. However, the authors found this relationship only in conjunction with the personality trait of extraversion. We did not survey extraversion because we were not interested in the effect of particular personality traits but rather on the influence of background music on inhibitory mechanisms in general.

### Electrophysiological Results

In the present work, we did not find differences in the two components of interest (N2, P3) as a function of different acoustic backgrounds. The amplitude of the NoGo-N2 is associated with a successful inhibition of a response, as already mentioned in the introduction (Falkenstein et al., 1999). We could not find a difference in amplitude of the N2d between the different conditions. We also did not reveal differences in the amplitude of the P3d, which is associated with working memory update (Folstein and Van Petten, 2008), categorization processes (Azizian et al., 2006), and most importantly action inhibition (Kropotov et al., 2016, 2017). Furthermore, we did not find any harmful effects of background music on the performance of the Go/NoGo task. Therefore, we suggest that background music has no harmful effect on the execution of moderately difficult cognitive control (as in our study), even if it lasts for a certain period of time. On the contrary, our two musical conditions showed a significant increase in personal-rated arousal, which is generally an appreciated effect and even more so when the task which is being executed is repetitive and therefore boring after a certain period of time.

Due to the fact that some studies revealed a detrimental effect of background music on several tasks (Jäncke et al., 1994; Furnham and Bradley, 1997), it is important to show that at least in this context one can listen to music without the music interfering with the task performance. The opposite assumption that performance would improve with music played in the background (Nantais and Schellenberg, 1999; Thompson et al., 2001) could also not be verified by our data. Nantais and Schellenberg (1999) used music and narrated short stories as stimuli. Their work showed that acoustic stimulation, using stories as well as music, leads to better performance compared to silence. This result could not be replicated in any of the examined parameters of the present study. Only an increased arousal was reported by the participants when background music was played. This result is in contrast to the findings of Thompson et al. (2001), who reported better performance with increased arousal and positive affect. On the other hand, we did not find any differences in the mood evaluation between the conditions. It may be that both parameters are necessary for a positive influence on performance, and an increased arousal alone is not sufficient for a positive effect. A meta-analysis by Kämpfe et al. (2011) revealed that the tempo of performed activities was influenced by the tempo of background music, and the authors suggested arousal as a mediator. In our study, the higher arousal induced by the musical pieces had no influence on the speed of performance as reflected by the reaction times. Another possible moderating variable could be habituation (Behne, 1999). This refers to the fact that that over time, people may become used to the omnipresence of background music in daily life and therefore its influence would decrease. However, Kämpfe et al. (2011) could not find such a systematic decline.

### Limitations and Outlook

One of the limitations of this study certainly lies in the circumstance that musical preference is very individual, and that the music was presented without any choice options to the participants, and this usually does not represent common listening habits. Also Krause et al. (2015) hypothesized that music which is chosen by the subject has a positive effect and leads to increased attention in contrast to provided music over which the subject has no control. Nantais and Schellenberg (1999) have shown that performance is closely tied to the preferences of the listeners. It would also be advisable for future studies to collect data on how music is listened to in everyday life, for example, the choice of devices and whether the music is selected deliberately and listened to actively or whether it is consumed passively in the background. This would be interesting in that there might be differences in the commitment that is shown toward the music (Krause et al., 2015).

Kämpfe et al. (2011) mentioned in their meta-analysis, that a uniform effect of background music could not be found. This fact is also mirrored by the inconsistent findings in the literature overall. According to the authors, a possible reason for this lies in the circumstance that the influence of background music was studied in a too global manner. Therefore, it is important to examine the influence of background music in more detail to disentangle the effect of background music on specific processes, such as was the case in our study. In addition, more studies which investigate subcomponents of cognitive control are needed, because they accompany all our activities in some way.

## Conclusion

Listening to background music had no effects on the performance of an inhibition task. We focused our attention on components of cognitive control (N2, P3) and on performance measures (reaction times, omission, and commission errors) during the completion of the VCPT. Studies such as this continue to gain importance due to the growing relevance of music in our culture and its almost natural implementation in many parts of our daily lives. For this reason, it is important to examine whether or not background music has any influence on a given main task. We can state that no negative influence of background music on task performance was found in the framework of a cognitive control task. Therefore, we assume that listening to music does not hamper the accomplishment of various tasks in daily life. This is at least true for tasks of moderate difficulty.

## Availability of Data and Materials

The raw data supporting the conclusions of this manuscript will be made available by the authors, without undue reservation, to any qualified researcher.

## Author Contributions

AB made contributions to the design and interpretations of the results, performed statistical analysis, and drafted the manuscript. SE made contributions to the design and interpretations of the results, and critically revised the manuscript. DK recorded the data, made contributions to the design and interpretations of the results, and critically revised the manuscript. CB made contributions to the interpretations of the results, and critically revised the manuscript. LJ made contributions to the design and interpretations of the results, and critically revised the manuscript. All the authors approved the final manuscript.

## Conflict of Interest Statement

The authors declare that the research was conducted in the absence of any commercial or financial relationships that could be construed as a potential conflict of interest.

## Abbreviations

EXC: exciting
NM: no music
RLX: relaxing
VCPT: visual continuous performance task
